# Supplementary material for: Dynamic changes of the Prf/Pto tomato resistance complex following effector recognition
Source: Nat Commun. 2023 May 4;14:2568. doi: 10.1038/s41467-023-38103-6 (PMC10160066; doi:10.1038/s41467-023-38103-6)
Supplement: Supplementary file 1 — Supplementary Information [file 41467_2023_38103_MOESM1_ESM.pdf]

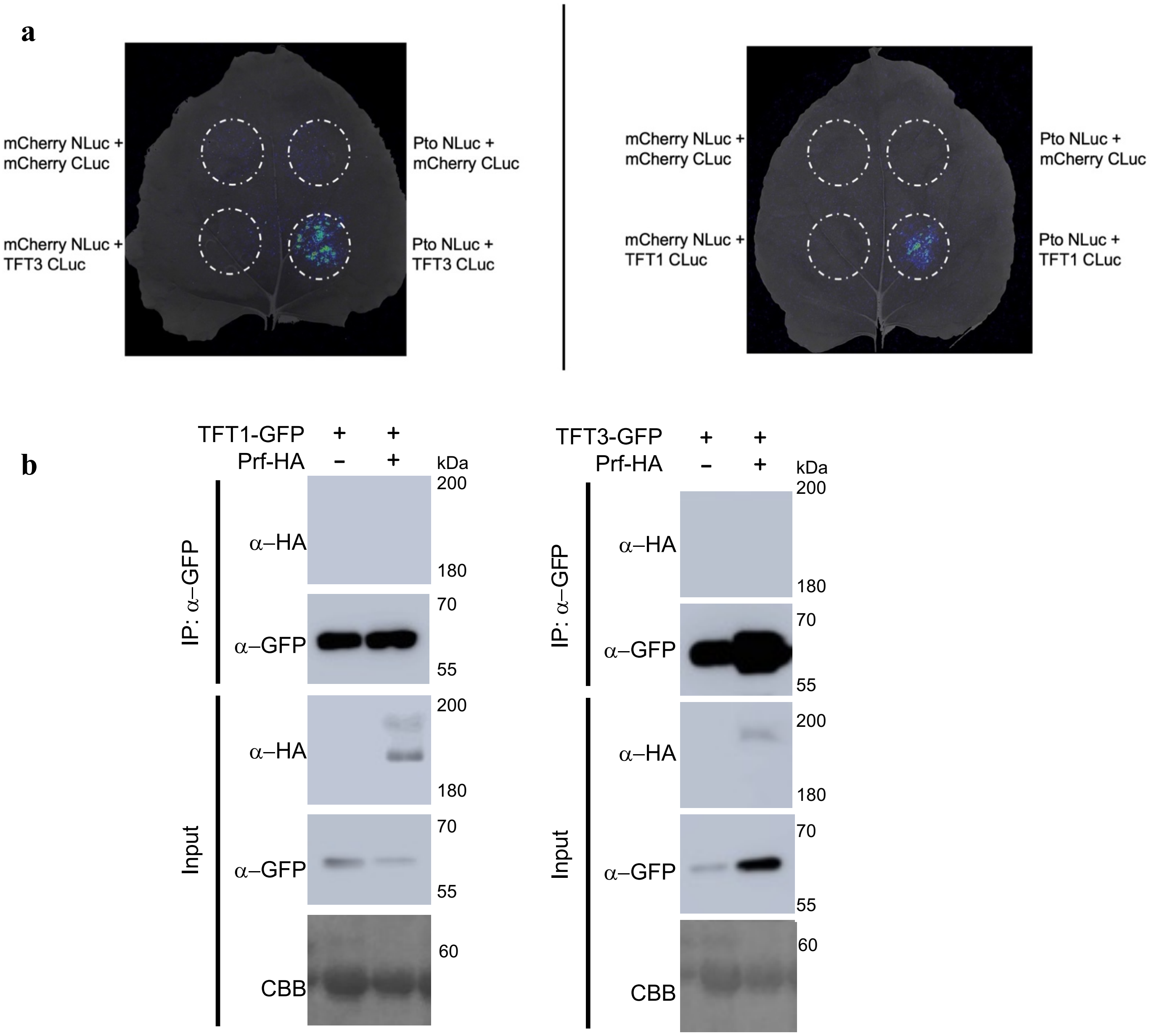

Supplementary Figure 1. Pto and not Prf interact with tomato 14-3-3 proteins.
a. Pto interacts with TFT3 and TFT1. Pto NLuc was transiently co-expressed with TFT3 CLuc (left) or TFT1 NLuc (right) along with the mCherry N/CLuc controls in *N. benthamiana* leaves. Leaf luminescence was detected 3 days post-infiltration using a Photek camera. The experiments were repeated three times and typical results are shown. **b.** Prf does not interact with tomato 14-3-3 proteins. Prf-HA was co-expressed with TFT1-GFP or TFT3-GFP in *N. benthamiana* leaves and TFTs were immunoprecipitated (IP) using GFP-Trap agarose beads. Plant leaves were harvested 3 days post infiltration for protein extraction and immunoblots were performed with the antibodies indicated on the left. Coomassie Brilliant Blue (CBB) staining of the membrane was used to monitor protein loading. The experiments were repeated three times and typical results are shown.

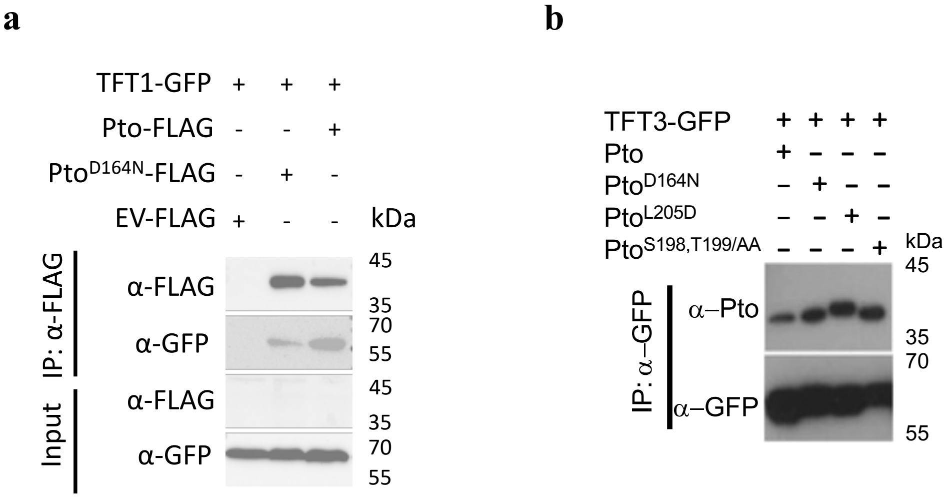

Supplementary Figure 2. The kinase activity of Pto is not required for its interaction with either TFT1 or TFT3.
a. TFT1-GFP, Pto-FLAG, EV-FLAG and the kinase dead variant of Pto (Pto^{D164N}-FLAG) constructs were transiently expressed in *N. benthamiana* leaves. Pto-FLAG and Pto^{D164N}-FLAG and EV-FLAG were immunoprecipitated (IP) using α-FLAG agarose beads. **b.** TFT3-GFP, Pto-FLAG, Pto^{D164N}-FLAG, the constitutive active Pto (Pto^{L205D}-FLAG) and transphosphorylation deficient Pto (Pto$^{S198,T199/AA}$-FLAG) constructs were transiently expressed in *N. benthamiana* leaves. TFT3-GFP was IP using GFP-Trap agarose beads. Leaves were harvested 3 days post infiltration for protein extraction and immunoblots were performed with the antibodies indicated on the left. The experiments were repeated three times and typical results are shown.

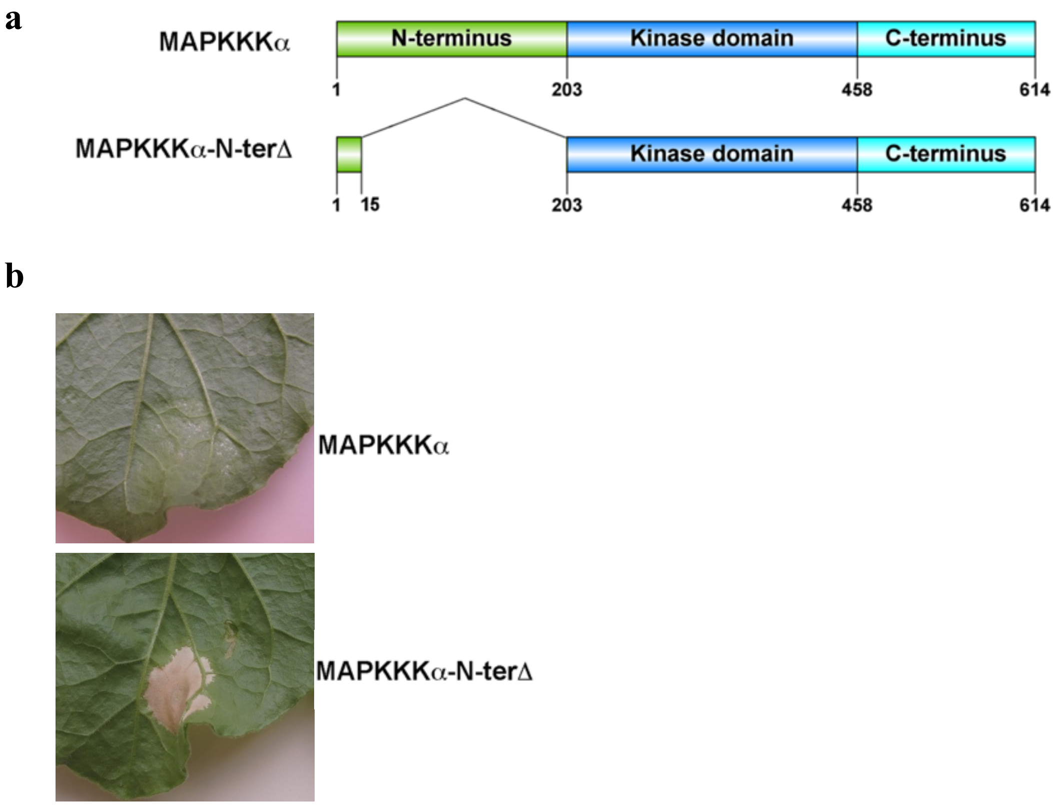

Supplementary Figure 3. Tomato MAPKKKα N-ter Δ is an autoactive version triggering ligand independent HR.

a. The N terminal deleted version of MAPKKKα lacking amino acids 16-202 (MAPKKKα N ter Δ) and full length MAPKKKα (**b**) were transiently expressed in *N. benthamiana* leaves and pictures were taken after 3 days.

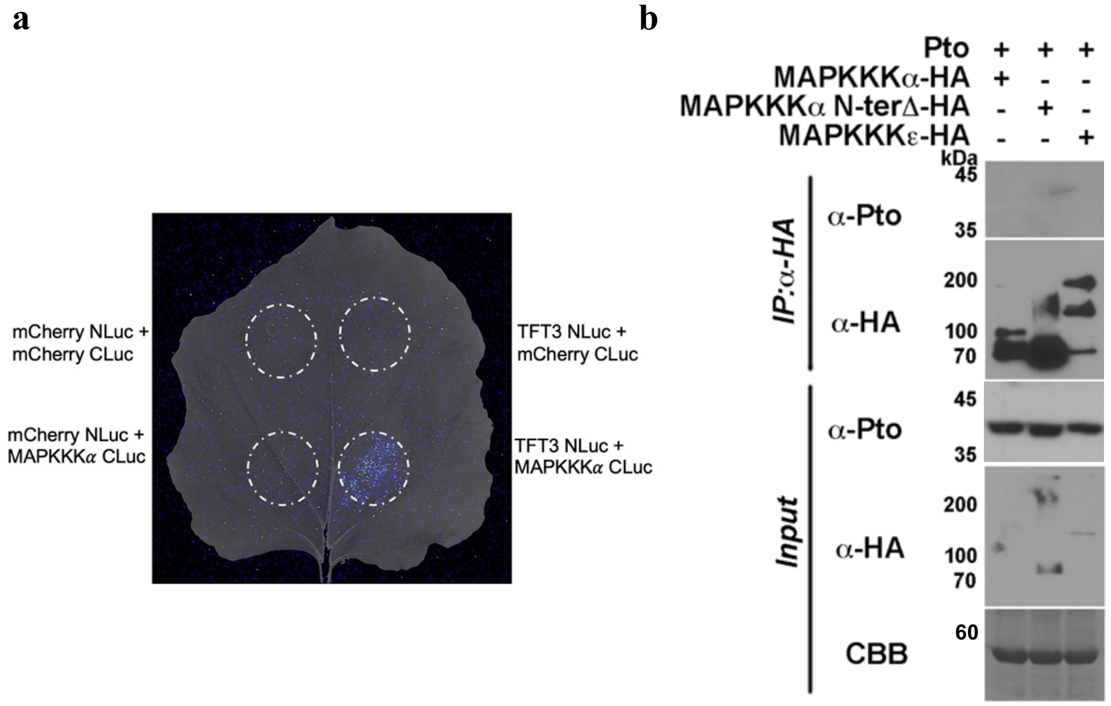

Supplementary Figure 4. TFT3 but not Pto interacts with MAPKKK*α*.

a. TFT3 interacts with MAPKKK*α*. TFT3 NLuc was transiently co-expressed with MAPKKK*α* CLuc along with the mCherry N/CLuc controls in *N. benthamiana* leaves. Leaf luminescence was detected 3 days post-infiltration using a Photek camera. The experiments were repeated three times and typical results are shown. **b.** Pto does not interact with MAPKKKs. HA-tagged MAPKKKα, MAPKKKε or the autoactive N-terminal deleted version of MAPKKKα (MAPKKKα N ter Δ) were co-expressed with Pto in *N. benthamiana* leaves as indicated and MAPKKKs were immunoprecipitated (IP) using α-HA agarose beads. Leaves were harvested 3 days post infiltration for protein extraction and immunoblots were performed with the antibodies indicated on the left. Coomassie Brilliant Blue (CBB) staining of the membrane was used to verify equal protein loading. The experiments were repeated three times and typical results are shown.

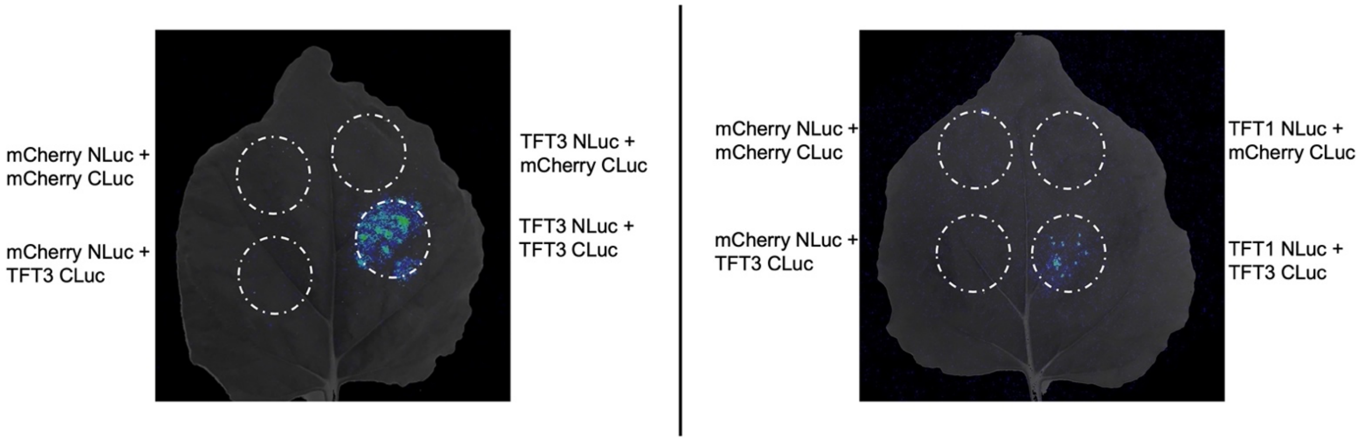

Supplementary Figure 5. TFT3 homo and hetero-dimerises with TFT1.
TFT3 NLuc was transiently co-expressed with TFT3 CLuc (left) or TFT1 (right) along with the mCherry N/CLuc controls in *N. benthamiana* leaves. Leaf luminescence was detected 3 days post-infiltration using a Photek camera. The experiments were repeated three times and typical results are shown.

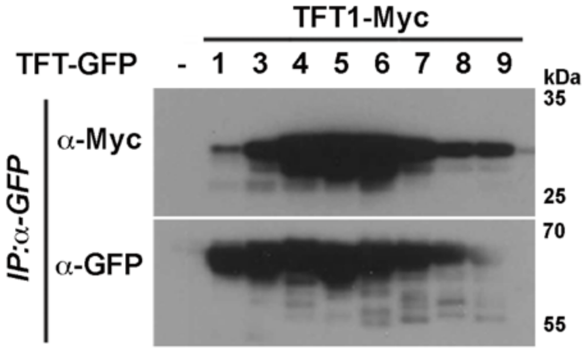

Supplementary Figure 6. TFT1 forms homo and hetero-dimers with multiple TFTs. TFT1-Myc was transiently co-expressed with TFT(1-9)-GFP in *N. benthamiana* leaves. Immunoprecipitation (IP) of TFT-GFP was carried out using GFP-Trap agarose beads. All leaves were harvest 3 days post-infiltration for protein extraction and immunoblots were performed with the antibodies indicated on the left. The experiments were repeated three times and typical results are shown.

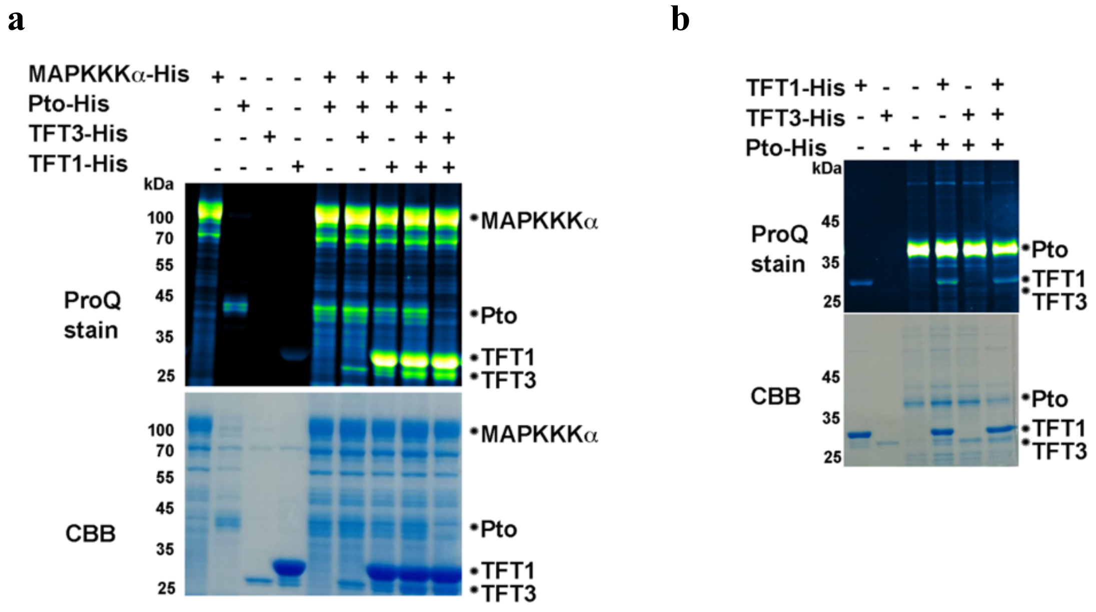

Supplementary Figure 7. Phosphorylation dynamics of Prf/Pto components.

a. Kinase activity assay showing the phosphorylation status of MAPKKKα *in vitro*. Purified His-tagged proteins (MAPKKKα, Pto, TFT1 and TFT3) were incubated in the indicated combinations in kinase buffer. The proteins were separated on a 10% SDS-PAGE gel and stained with ProQ Diamond stain which specifically stains phosphorylated proteins (bright green/blue bands). Coomassie Brilliant Blue (CBB) staining of the SDS-PAGE gels was used to verify protein loading. **b.** No direct phosphorylation of TFTs by Pto was observed. His-purified Pto, TFT1 and TFT3 were incubated in a kinase buffer. Proteins were separated on a 10% SDS-PAGE gel and stained with ProQ Diamond stain. CBB staining of the SDS-PAGE gels was used to verify protein loading. All experiments were performed 3 times and typical results are shown.

a

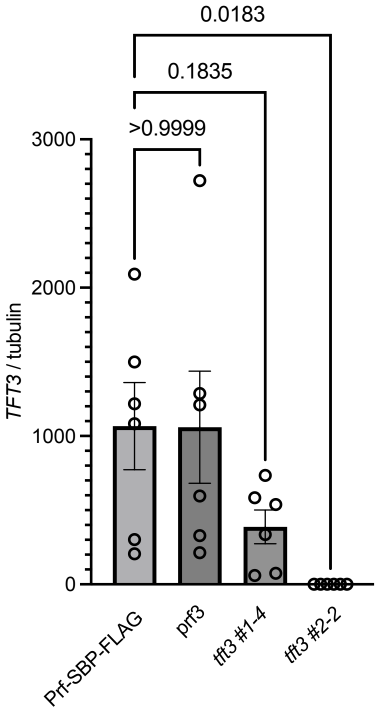

b

```
TFT3

ATGGCGGTGGCACCGACGGCGCGTGAGGAGAACGTGTACATGGCAAAGCTGGCGGAGCAGGCCGAGAGGTATGAAGAGATGGTG
 M  A  V  A  P  T  A  R  E  E  N  V  Y  M  A  K  L  A  E  Q  A  E  R  Y  E  E  M  V

GAATTCATGGAGAAGGTCTCCAACTCCCTTGGCTCGGAAGAACTAACCGTGGAGGAGAGAAACCTTCTCTCCGTCGCGTATAAG
 E  F  M  E  K  V  S  N  S  L  G  S  E  E  L  T  V  E  E  R  N  L  L  S  V  A  Y  K
                   Target       PAM
AATGTGATCGGAGCGCGTAGGGCATCATGGCGTATCATTTCATCGATCGAGCAAAAGGAAGAGTCTAGAGGAAATGAGGAACAT
 N  V  I  G  A  R  R  A  S  W  R  I  I  S  S  I  E  Q  K  E  E  S  R  G  N  E  E  H

GTTAACTCTATCCGCGAGTACAGATCTAAGATTGAGA   (wild-type)
 V  N  S  I  R  E  Y  R  S  K  I  E


tft3 #2-2

ATGGCGGTGGCACCGACGGCGCGTGA----------------------------------------------------------
 M  A  V  A  P  T  A  R  D
------------------------------------------------------------------------------------

------------------------------------------------------------------------------------

------------------------TCTAAGATTGAGA   (250bp deletion)
                          L  R  L  R
```

Supplementary Figure 8. Expression level of *TFT3* following infection with *Pst* DC3000. **a.** qPCR analyses of *TFT3* expression in Rio Grande Tomato lines *prf3*, *prf3*/Prf-SBP-FLAG (Prf-SBP-FLAG), *prf3/tft3 1-4*/Prf-SBP-FLAG (*tft3 #1-4*) and *prf3/tft3 2-2*/Prf-SBP-FLAG (*tft3 #2-2*). Relative expression levels were calculated compared to tubulin expression values and are means ± SE, n = 3. Significance was calculated using multiple two-sided T-tests where * shows significance according to the indicated *P*-values compared to Prf-SBP-FLAG expression. The experiment was repeated three times and typical results are shown. **b.** Generation of TFT3 null mutant. TFT3 mutant line, *tft3* #2-2, generated by CRISPR/Cas9 system in Rio Grande tomato line *prf3*/Prf-SBP-FLAG plant. Red and blue letters represent CRISPR/Cas9 target and PAM (Protospacer Adjacent Motif) sequences, respectively. Black dashes represent the deletions. Translated protein sequences are represented under the DNA sequences.

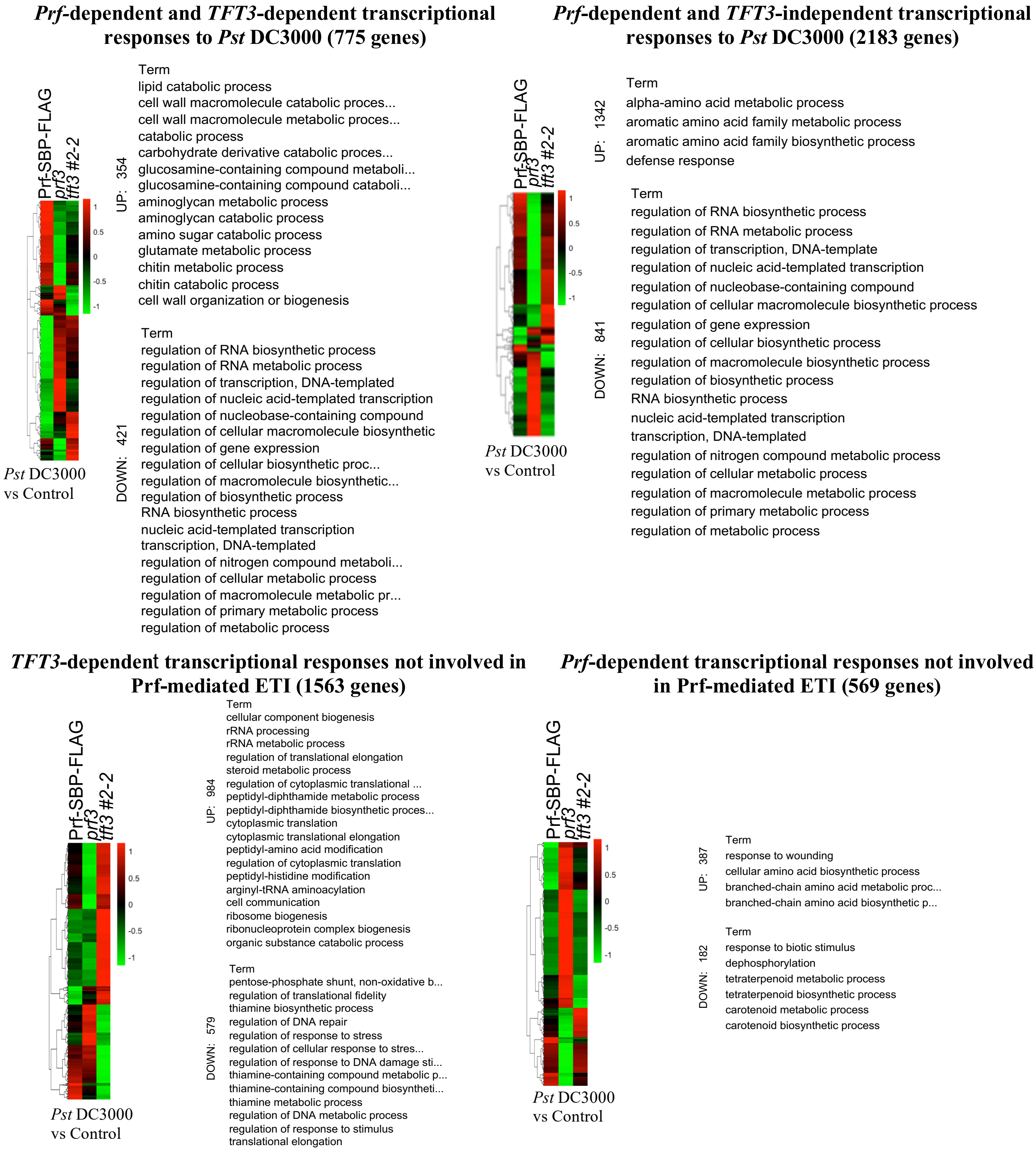

Supplementary Figure 9. Prf and TFT3 regulated distinct and overlapping sets of genes. Heat maps and gene ontology (GO) enrichment analysis showing the differential expression profile of wild type and mutant tomato lines 6 hours post infiltration with *Pseudomonas syringae* pv. *tomato* (*Pst* DC3000). Rio Grande tomato lines, *prf3*, *prf3*/Prf-SBP-FLAG (Prf-SBP-FLAG) and *prf3/tft3 2-2*/Prf-SBP-FLAG (*tft3 #2-2*) were either mock inoculated (control) or infiltrated with *Pst* DC3000. Threshold was set at greater than 2-fold and transcripts were termed to be DEGs if they showed a Benjamini-Hochberg adjusted P-value ≤ 0.05 in the comparison between treatment and control. GO term enrichment was analysed with Fisher Exact test, false discovery rate (FDR) < 0.05. Gene expression changes are coloured depending on whether genes are relatively induced (red) or repressed (green) within infected leaves compared to mock-inoculated leaves. All infiltrations were carried out in biological triplicates.

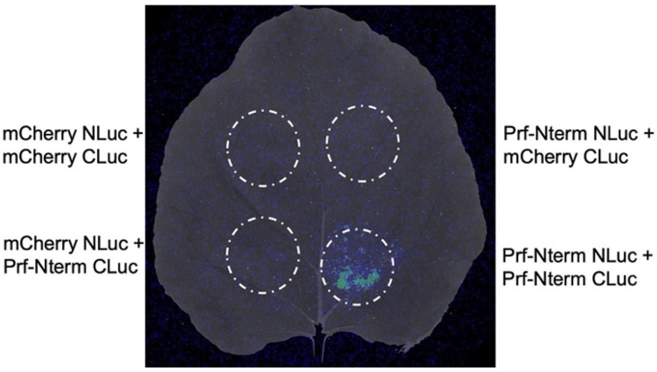

Supplementary Figure 10. The Prf N-term domain self-associates.
Prf-Nterm Nluc was transiently co-expressed with Prf-Nterm CLuc along with the mCherry N/CLuc controls in *N. benthamiana* leaves. Leaf luminescence was detected 3 days post-infiltration using a Photek camera. The experiments were repeated three times and typical results are shown.

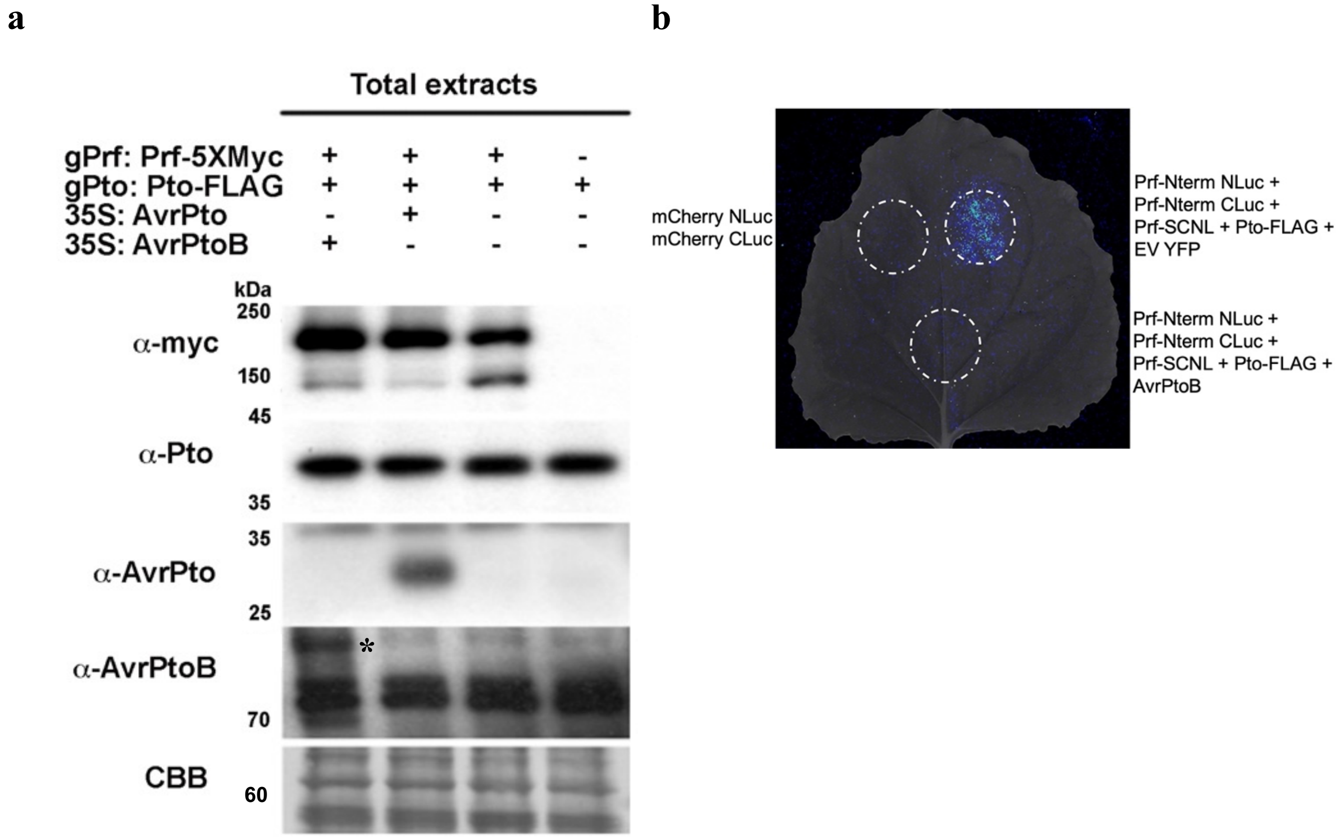

Supplementary Figure 11. The Prf-Prf dimer dissociates after effector recognition.
a. Expression confirmation of individual proteins for Prf/Pto complex on Blue Native PAGE**.** Prf-Myc and Pto-FLAG constructs were transiently expressed from their native promoters in *N. benthamiana* leaves in the presence or absence of AvrPto or AvrPtoB. Prior to immunoprecipitation and blue native PAGE, equal protein levels were confirmed using immunoblotting with the antibodies indicated on the left (*indicates the AvrPtoB specific band). All plant leaves were harvested 3 days post infiltration for protein extraction. Coomassie Brilliant Blue (CBB) staining of the membrane was used to verify protein loading. The experiments were repeated three times and typical results are shown. **b.** Prf homodimers dissociates after AvrPtoB recognition. Prf-Nterm NLuc was transiently co-expressed with Prf-Nterm CLuc along with Prf-SCNL, Pto-FLAG and the mCherry N/CLuc controls in *N. benthamiana* leaves, in the presence or absence of AvrPtoB. Leaf luminescence was detected 3 days post-infiltration using a Photek camera. The experiments were repeated three times and typical results are shown.

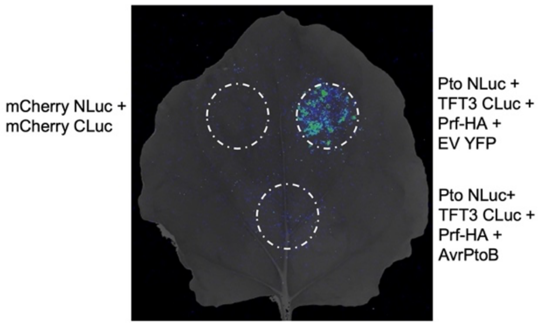

Supplementary Figure 12. TFT3 and Pto dissociate after effector recognition. Pto NLuc was transiently co-expressed with TFT3 CLuc along with Prf-HA and the mCherry N/CLuc controls in *N. benthamiana* leaves, in the presence or absence of AvrPtoB. Leaf luminescence was detected 3 days post-infiltration using a Photek camera. The experiments were repeated three times and typical results are shown.

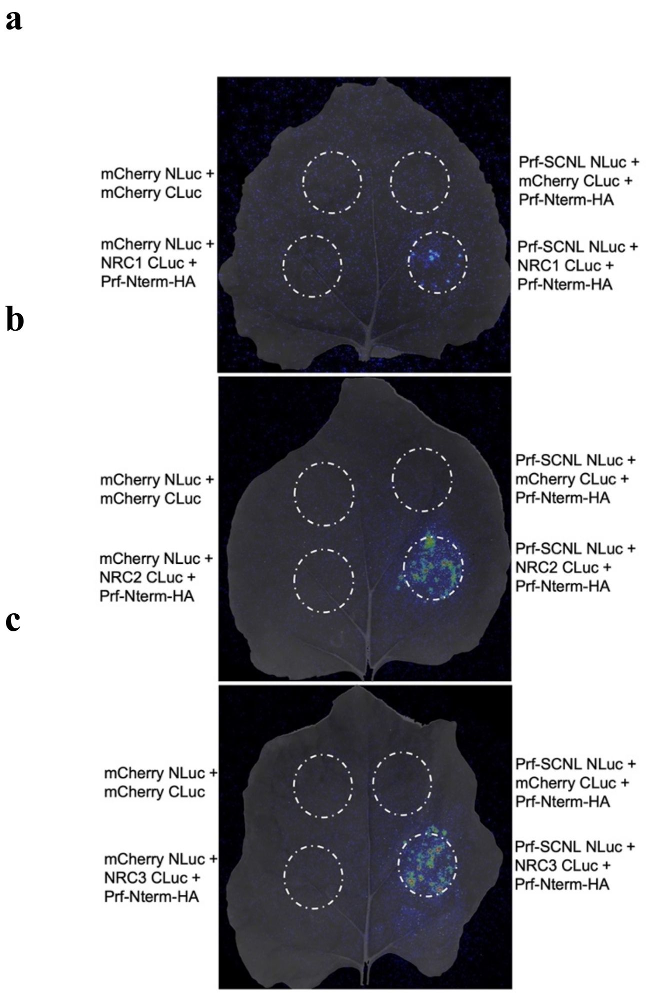

Supplementary Figure 13. NRCs interact with Prf.
Prf-SCNL NLuc was transiently co-expressed with NRC1 (a), NRC2 (b) or NRC3 (c) CLuc along with Prf-Nterm-HA and the mCherry N/CLuc controls in *N. benthamiana* leaves. Leaf luminescence was detected 3 days post-infiltration using a Photek camera. The experiments were repeated three times and typical results are shown.

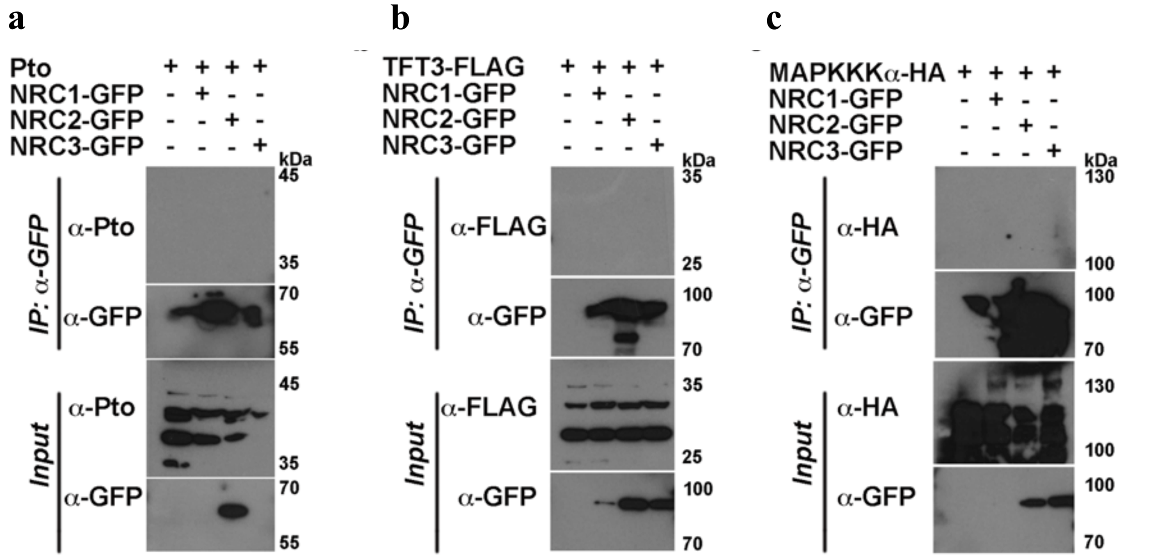

Supplementary Figure 14. NRCs do not interact with Pto (a), TFT3 (b) or MAPKKKα (c). **a-c** Pto, TFT3-FLAG, MAPKKKα-HA and NRC1-3-GFP were transiently expressed in *N. benthamiana* leaves. NRCs were immunoprecipitated (IP) using GFP-Trap agarose beads. Plant leaves were harvested 3 days post infiltration for protein extraction and immunoblots were performed with the antibodies indicated on the left of each panel. The experiments were repeated three times and typical results are shown.

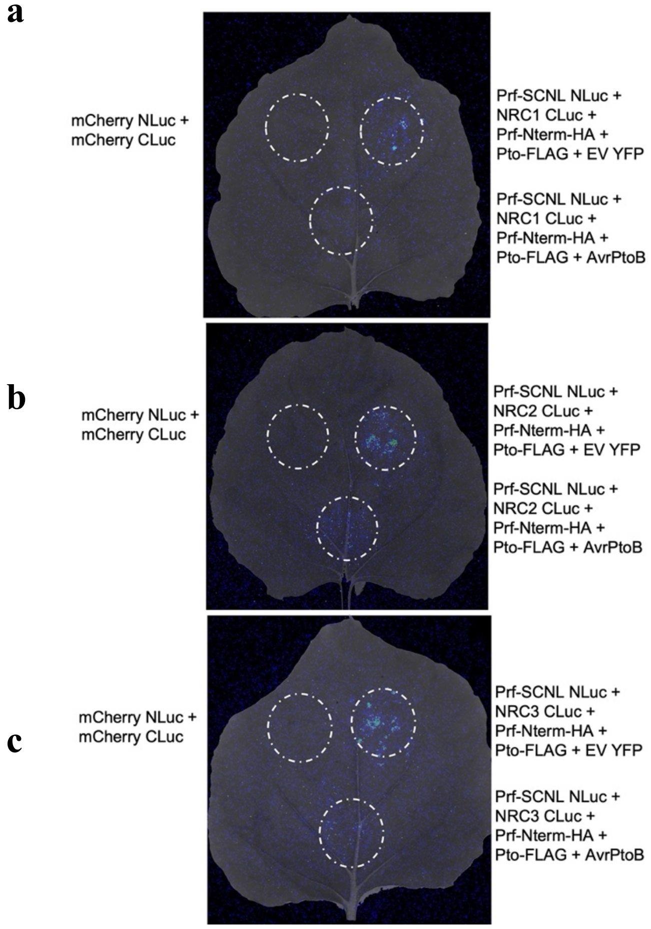

Supplementary Figure 15. NRCs and Prf dissociate after effector recognition. Prf-SCNL NLuc was transiently co-expressed with NRC1 (a), NRC2 (b) or NRC3 (c) CLuc along with Prf-Nterm-HA, Pto-FLAG and the mCherry N/CLuc controls in *N. benthamiana* leaves, in the presence or absence of AvrPtoB. Leaf luminescence was detected 3 days post-infiltration using a Photek camera. The experiments were repeated three times and typical results are shown.

Supplementary Table 1. Primers used

Gene Name	Forward Primer	Reverse primer	Purpose
TFT1	AAAAAGCAGGCTCCACCATGGCCTTGCCTGAAAATTT	AGAAAGCTGGGTCAGCCTCGTCCATCTGCTC	Cloning in pDONR-Zeo vector
TFT2	AAAAAGCAGGCTCCACCATGGCGCGTGAGGAG	AGAAAGCTGGGTC CTG TTG TTC ATT GTCG	
TFT3	AAAAAGCAGGCTCCACCATGGAGAAGGTCTCCAACTC	AGAAAGCTGGGTCATTTTTCTCTTCAGGTTTGG	
TFT4	AAAAAGCAGGCTCCACCATGGCTGACTCTTCGCG	AGA AAG CTG GGT CCTG CTG CCC CTC GCC T	
TFT5	AAAAAGCAGGCTCCACCATGGCTCTCCACGTGAA	AGAAAGCTGGGTCTCCTGCATATCCGAGGT	
TFT6	AAAAAGCAGGCTCCACCATGGCGTCGCCACGC	AGA AAG CTG GGT CTTCATTATCATC TGG TTT AGG	
TFT7	AAAAAGCAGGCTCCACCATGGAGAAGGAAAGAGAAAAACA	AGAAAGCTGGGTCGTTCTCTCCCTGGCGCTC	
TFT8	AAAAAGCAGGCTCCACCATGGCTTCATCCAAAGAACG	AGAAAGCTGGGTC CTC CGC ATC CTC GCC T	
TFT9	AAAAAGCAGGCTCCACCATGGCT TCTTCCAAAGAACG	AGA AAG CTG GGT CCTCTGCATCTTC ACC TCC A	
TFT10	AAAAAGCAGGCTCCACCATGGCGGCTCTAATCCC	AGAAAGCTGGGTCAGATTCATCCAACTGATC C	
Pto	AAAAAGCAGGCTCCACCATGGGAAGCAAG	AGAAAGCTGGGTCAATAACAGACTCTTG	
Prf-SCNL	GGGGACAAGTTTGTACAAAAAAGCAGGCTCTATGATTCCCAAGATGGATGAGATA	GGGGACCACTTTGTACAAGAAAGCTGGGTCTAAGAGTCAAGGGGCTGTTC	
SlMAPKKKα	GGGGACAAGTTTGTACAAAAAAGCAGGCTATGCCTGCTTGGTGGGGAAA	GGGGACCACTTTGTACAAGAAAGCTGGGTTTCTTCGGTGCATGCAAAAA	
α-Tubulin	CCAATCTCAATCGCCTTAT	CACCACATCACCACGGAAC	qRT PCR
TFT3	CCTTGGCTCGGAAGAACTAAC	CAGATGTTGCTGAAGGAATAAG	
sgRNArev/TFTtarget	GTTTTAGAGCTAGAAATAGCAAGTTAAAAT	TGATGCCCTACGCGCTCCGACAATCCTACTTCGACTCTAGCTG	CRISPR/Cas9
TFTfor/TFTrev	ATGGCGGTGGCACCGACGGC	GCAATTTGAGAATGCCATCA	
