# Supplementary material for: Dynamic changes of the Prf/Pto tomato resistance complex following effector recognition
Source: Nat Commun. 2023 May 4;14:2568. doi: 10.1038/s41467-023-38103-6 (PMC10160066; doi:10.1038/s41467-023-38103-6)
Supplement: Supplementary file 3 — Description of Additional Supplementary Files [file 41467_2023_38103_MOESM3_ESM.pdf]

Description of Additional Supplementary Files:

Supplementary Data 1: ETI transcriptional responses are compromised in tomato tft3 and prf3 mutants.
